# Effect of work on body language of ranch horses in Brazil

**DOI:** 10.1371/journal.pone.0228130

**Published:** 2020-01-28

**Authors:** Pedro Henrique Esteves Trindade, Elke Hartmann, Linda J. Keeling, Pia Haubro Andersen, Guilherme de Camargo Ferraz, Mateus José Rodrigues Paranhos da Costa

**Affiliations:** 1 Graduate Program in Animal Science, Faculty of Agricultural and Veterinary Sciences, UNESP, São Paulo State University, Jaboticabal-SP, Brazil; 2 Department of Animal Science, Ethology and Animal Ecology Research Group (Grupo ETCO), Faculty of Agricultural and Veterinary Sciences, UNESP, São Paulo State University, Jaboticabal-SP, Brazil; 3 Departament of Animal Environment and Health, Swedish University of Agricultural Sciences, Uppsala, Sweden; 4 Departament of Large Animal Sciences, University of Agricultural Sciences, Uppsala, Sweden; 5 Departament of Animal Morphology and Physiology, UNESP, São Paulo State University, Faculty of Agricultural and Veterinary Sciences, Jaboticabal-SP, Brazil; University of Illinois, UNITED STATES

## Abstract

The horses’ responses to exercise are commonly monitored using physiological variables, nonetheless physical and mental states can also be expressed through body language. The aims of this study were: (*i*) to identify how facial expressions and other behavioural variables change in ranch horses after a routine workday, and (*ii*) to investigate if these changes can be used as indicators of physical tiredness by relating them to known variables of physical fitness and workload. Fourteen crossbred ranch horses were assessed pre- and post-workday on two farms, recording the body language, physiological and workload variables. Statistical analysis consisted of four stages: (*i*) comparisons between the sampling times (pre- *vs* post-workday) using linear mixed-effects models with repeated measures and a paired Wilcoxon test; (*ii*) selection of the most powerful variables by applying Kaiser-Meyer-Olkin test and principal components analyses (PCA); (*iii*) evaluations of the relationships within these selected variables utilizing PCA and Spearman rank coefficients; and (*iv*) identifying a critical level of the most robust behavioural indicators using a non-hierarchical cluster analysis. Results showed that after a workday the horses increased the frequency/duration of body language indicative of resting. They also decreased the frequency/duration of body language indicative of attention and movements to avoid flies. However, some of these behaviours are also shown when horses are in pain, leading us to suggest that some ranch horses were probably experiencing a combination of of tiredness and slight soreness. Of particular interest, because of the ease with which it can be assessed on the farm and generalized to other situations, we suggest that the frequency of shifting weight between the forelegs has potential to be used as an indicator of physical tiredness in horses. The results can also be used in the development of tools to improve the welfare of ranch horses as well as horses used in other activities, although more research is needed to validate this assumption.

## Introduction

Around 72% of approximately 5 million horses in Brazil are on beef cattle farms [1], being used mainly for cattle driving and for moving around on the farm to check the condition of the animals, pastures, fences and water troughs [2]. Horses have been used for ranch work since the XVI century in Brazil, and the possibility of replacing them by machines is often considered infeasible, due to economic or technical reasons [2].

Although ranch horses have an important role in Brazilian beef farms, almost nothing is known about the welfare of these animals. For instance, despite it being common to observe long periods of daily work, we found only one study assessing the "physiological adaptations of the Pantaneiro horse to stress related to daily work", and this was done by measuring only respiratory and heart rates [3]. Long and intense workdays could result in muscle fatigue, rhabdomyolysis, exhaustive disease syndrome, as well as overtraining [4, 5] that can compromise the ranch horses’ welfare. For this reason, the intensity of the exercise performed by ranch horses and the horse’s physical response to the work should be investigated more thoroughly, hence the current study.

Assessment of ranch horses’ welfare can also be based upon their body language, that is to say, their body posture and movements (other behaviours), and even their facial tension and movements (facial expressions), all of which allow us to identify if the animals are in stressful or painful states. This approach has the advantages of being non-invasive and easy to apply in the field [6–9]. For example, in most behaviourally based pain scales for horses, agitation and a low head carriage are considered nonspecific behaviours indicative of acute pain, whereas weight-shifting between forelegs, resting hindlegs and lameness can be considered indicators for limb and foot pain [10–12]. Facial expressions have also been used to assess pain and other emotional states in many animal species, such as mice [13, 14], rabbits [15], dairy cattle [16], pigs [17, 18], sheep [19, 20] and horses [21–26]. However, we did not find any study of facial expressions, or body posture and movements associated with physical tiredness in horses.

The aims of this study were (*i*) to identify how facial expressions and other behavioural variables change in ranch horses after a routine workday, and (*ii*) to investigate if these changes can be used as indicators of physical tiredness in ranch horses by relating them to known variables of physical fitness (metabolic, endocrine and biochemical responses to exercise) and workload (distance, duration and average velocity). We hypothesized that there will be changes in facial expressions and other behaviours after a workday, and that some of these will correlate sufficiently well with the amount of exercise performed by the horses to be used as indicators of physical tiredness in ranch horses.

## Material and methods

This research was authorized by the Ethics Committee for the Use of Animals from the Faculty of Agricultural and Veterinary Sciences, University of São Paulo State, protocol number 13466/15.

### Animals and management

Fourteen crossbreed ranch horses (13 geldings and 1 mare), with moderate or good body condition, physically fit and clinically healthy (evaluated by physical examination and blood analysis, see S1 Appendix), and ranging in age from 6 to 27 years (mean±SD: 14.5±5.1 years) were used in this study. The ranch horses were kept at two commercial beef cattle farms, Farm 1 (F1: n = 6) and Farm 2 (F2: n = 8), both located in the municipality of Sertãozinho (approximately 579 m altitude), São Paulo State, Brazil, and presenting similar topography, with gently sloping terrains.

The horses were usually used for checking cattle, driving them and for verifying pasture, fence and water trough conditions. The Brazilian cowboys usually do their daily work in pairs or in groups of three, and when working on foot the horses are often tied up at a post, still wearing their saddle and bridle. When not being ridden, horses were kept in groups (minimum two horses) on pasture with *ad libitum* grass (*Paspalum notatum* and *Cynodon dactylon* at F1; and *Cynodon dactylon* at F2), water and a mineral supplement. Additionally, the horses received supplementary feed consisting of 1 kg corn, 1 kg soybean, 800 g calcium carbonate per horse/day at F1, and at F2 the horses received 2 kg of commercial feed concentrate per horse/day. None of the horses wore horseshoes.

### Data collection

Data were recorded during seven non-continuous days in the summer, from December 2015 to January 2016, following the work routine of each farm. The horses were always evaluated in pairs during routine working days, and each horse acted as its own control. Ten horses worked a half day (eight from F2 and two from F1, always in the morning; mean ± SD: 2.9 ± 0.4 hours) and the other four horses (all from F1) worked the entire day (morning and afternoon; mean ± SD: 7.9 ± 1.0 hours). The horses that worked the entire day had around one hour of “resting” period during lunchtime, when they were kept in a paddock, keeping their saddle on their back.

The behavioural and physiological assessments started after a resting period (without being used for work in the farms) of at least 12 hours. Each pair of horses was assessed both prior to a workday (pre-workday, baseline) and after a regular workday (post-workday), being ridden always by the stockperson who usually rode them. Before starting the workday, a digital heart rate monitor (Garmim Forerunner 310XT) was fitted on each horse. To minimize the disturbances to commercial farm routines, pre-workday assessments were carried out twice; one day before (approximately 14 h before starting work) and then 40 to 50 min before the start of the routine workday, while post-workday assessments were carried out three times, immediately after work and again, 6 and 12 h after finishing the work (see Table 1 for details). The lactate, creatine kinase and aspartate aminotransferase sampling times were selected to target the concentration peak after exercise according to other studies, being assessed immediataly after workday, and between 15 to 30 min. [27], 6–12 hours, and 12–24 hours after a workday [9]. The post-workday samples were collected around 11:30 to 12:30 or 16:30 to 17:30h, to be compared with the samples before exercise, which were taken at 17:30 to 18:30h the previous day. Thus, all blood samples were collected out of the period of plasma cortisol concentration peak, which occurs early in the morning [28].

**Table 1:** Overview, in chronological order, of the data collection in ranch horses during the pre- and post-workday assessments.

Period		Time	Data collection	Assessments
Pre-workday assessments	Day before	Between 17:30 and 18:30 h	Clinical examination	Body condition score and lameness assessment
			Blood sample	Total protein, cortisol, lactate, glucose, creatine kinase and aspartate aminotransferase
	Before the start of the routine workday	Between 06:30 and 08:30 h, i.e. 40–50 min before workday	Clinical examination	Lameness assessment
			Respiratory rate—Direct observation	Counting flank/nostril movements for 15 s
			Other behaviours—video record	5 min filming of horse’s body
			Face expressions—video record	5 min filming of horse’s face
Post-workday assessments	Immediately after work	At the end of the workday	Clinical examination	Lameness assessment
		Immediately after lameness assessment post-workday	Respiratory rate—direct observation	Counting flank/nostril movements for 15 s
		Immediately post-workday	Blood sample	Total protein, cortisol, lactate, glucose, creatine kinase and aspartate aminotransferase
		3 min post-workday	Other behaviours—video record	5 min filming of horse’s body
		10–12 min post-workday	Face expressions—video record	5 min filming of horse’s face
		15 min post-workday	Blood sample	Lactate and glucose
		30 min post-workday	Blood sample	Lactate and glucose
		60 min post-workday	Blood sample	Lactate and glucose
		75 min post-workday	Clinical examination	Lameness assessment
	Later after work	6 h post-workday	Blood sample	Creatine kinase and aspartate aminotransferase
		12 h post-workday	Blood sample	Creatine kinase and aspartate aminotransferase

On both farms, pre- and post-workday data collections were carried out with the horses tied up near the saddling facilities. For this study, after the post-workday assessment the horses were kept in pairs in a paddock (around 1,600 m^2^) or in single pens (99 m^2^) until data collection had been completed (12 hours post-workday), returning to their pasture afterwards. Air temperature and humidity were measured before the start of each workday and immediately after work, and the means±SDs were 25.6±1.4 and 29.3±1 °C, and 74.4±6.5 and 68.9±5.6%, respectively.

### Body language categories and variables

The body language of undisturbed horses was recorded with a video camera (HDR-CX240, Sony^®^, Brazil) for 10 min. A recording consisted of 5 min to record their facial expressions (the face in profile due to the ease of evaluating its features) and another 5 min to record other behaviours (whole body view). The camera was placed approximately 2 m from the horse to assess facial expressions and 3 m to assess the other behaviours. The videos were always recorded in the same place at each farm. The horses were fitted with a halter and tied up on a loose rope, without the saddle during pre-workday assessment and with the saddle during post-workday recordings.

### Facial expression categories and variables

First, all video recordings were watched twice (normal and high speed) by the same observer to build an ethogram, including descriptions of facial expressions, which could be considered useful indicators to assess physical tiredness in ranch horses. Additional descriptions of facial muscle tension and movement were selected from ethograms in previous studies on the facial expressions in horses (e.g. Equine Facial Action Coding System [EquiFACS] [29]) or from studies that used these expressions as indicators of pain [22, 24–26].

**Fig 1:**
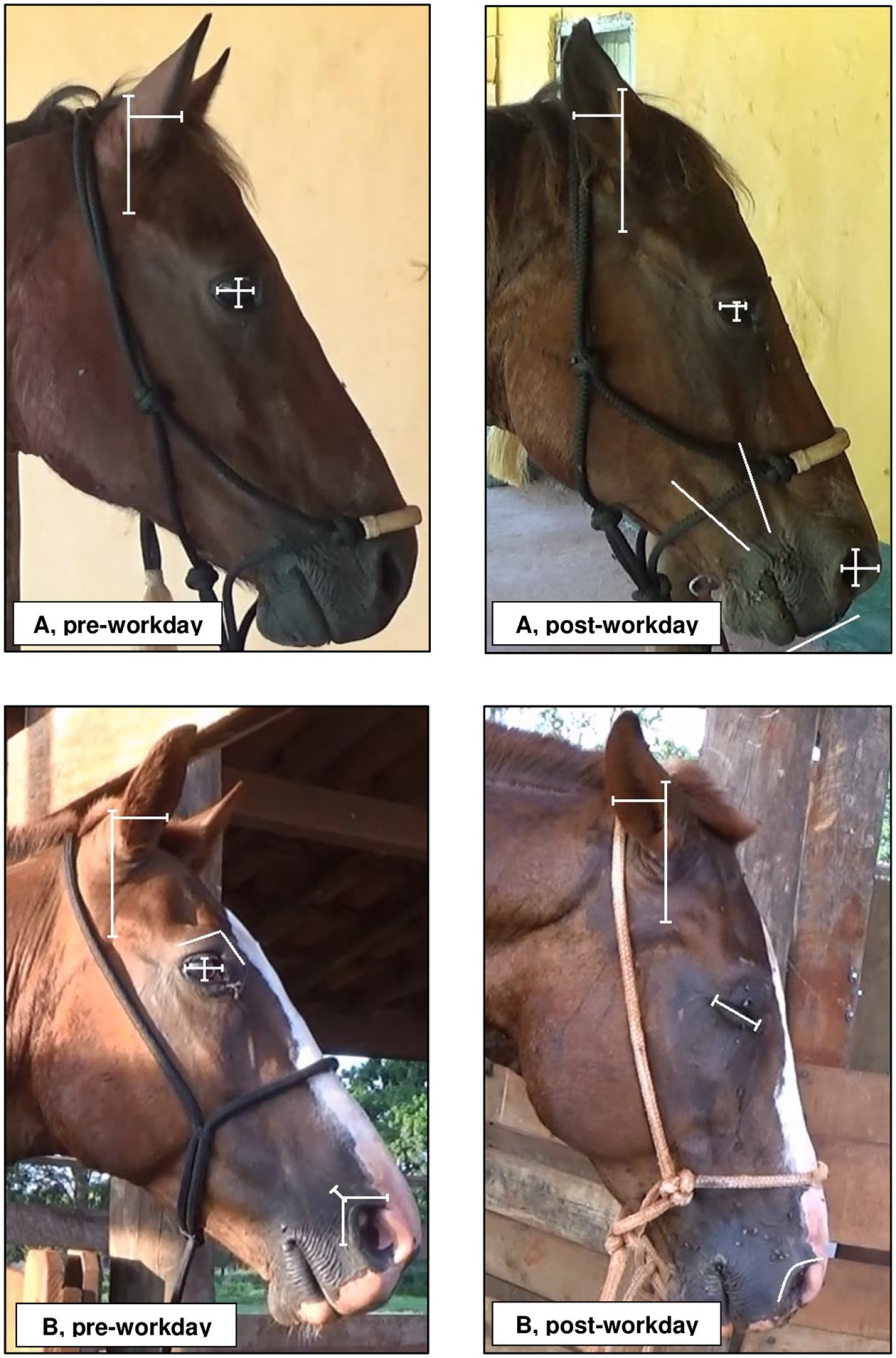
Still images from 30-s video clips of two ranch horses (A and B) pre- and post-workday. The images show both ranch horses orienting their ears forward and backward before and after workday, respectively. Horse A has its eye partially closed (A, post-workday), while horse B has its eye fully closed and is showing contraction of the eyelid post-workday. Horse A showed contraction of the inner brow raiser before and after the workday, and showed tension of the muzzle and mimic muscles post-workday. Horse B showed nostril lift before workday. See Table 2 for descriptions of the categories of facial expressions.

Two 30-s video clips were extracted from the 5-min face recording of each horse on the pre- and post-workdays for analysis of the facial expressions. These 30-s video clips were randomly chosen with the constraint that there was good light, good profile, and minimal external disturbances to give an accurate/clear view of the head/face of the horse [24]. The 30-s video clips were transferred to an annotation software (ELAN, ELAN Linguistic Annotator, version 4.9.4). Each 30-s video clip was observed 12 times using normal and slow-motion speeds (depending on what was being coded on that particular occasion) in order to accurately collect all facial expression categories. The scores, frequencies and/or durations of each category of facial expressions (see Table 2 and Fig 1) were recorded using continuous recording and animal focal sampling methods [30]. We recorded the duration of ear orientation at each position (forward, midline, backward and flattened) for each side (right and left), and after that we summed the values from each side creating a total duration of both ears at each position [24]. For data analysis, the values of frequencies and durations from the two 30-s video clips of the same horse and assessment (pre- or post-workday) were summed.

**Table 2:** Ethogram of the 18 facial expressions variables (adapted from [22, 24, 26, 29]) and their respective recording method (F = frequency, D = duration, S = score; continuous sampling).

Categories of facial expressions	EquiFACS Codes	Descriptions
Ears forward [22,24,29]	EAD101	Both ears are turned or swivelled forward (rostrally). D
Ears midline [22,24,29]	EAD102	both ears are pulled towards the midline (i.e. adducted). D
Ears flattener [29]	EAD103	both ears are flattened and abducted. D
Ears backward [22,24,29]	EAD104	both ears are rotated laterally and dorsal/caudally. The opening of the inner ear is turned outwards. D
Inner brow raiser [24,29]	AU101	The skin above the inner corner of the eye is pulled dorsally and obliquely towards the medial frontal region. D
Eye fully closed [22,29]	AU143	The upper and lower eyelids move towards each other to close the eye. Distinguishing when the eyelids meet, and the eye is only closed, and the horse exhibits some tension in the skin covering and surrounding the eye after it closed its eye. D
Eye partially closed [22,26,29]	AU47	Reduction of the eye opening by the eyelids drawing the eyelids or the skin around the eye contracting. Although the opening of the eye is reduced, the eye does not close completely. F and D
Eye white showing [26,29]	AD1	The white sclera becomes visible, or if present at rest there is an increase in the amount noticeable. F
Blink lower [29]	AU143	The upper and lower eyelids move towards each other to close the eye, and the eyes close for more than half a second. F
Blink fast [29]	AU145	The upper and lower eyelids move towards each other to close the eye, and the eyes close for less than half a second. F
Contraction of the orbicularis [26,29]	--	Contraction of the orbicularis muscle. F
Lower lip relaxed [26,29]	AU160	The lower lip is visibly relaxed and hangs loose with no tension. D
Lower lip depressor [26,29]	AU16	The lower lip is pulled down ventrally. F
Upper lip raiser [26,29]	AU10	The centre of the upper lip is raised straight up, and in exteme cases the rest of the upper lip may also be partially raised. F
Nostril lift [29]	AUH13	The caudal (back) edge of the nostril is pulled up and drawn round laterally. D
Tension of the muzzle [24,26]	--	There is increased tonus of the lips and tension of the chin resulting in an edged shape of the muzzle. When a horse showed tension of the muzzle in both 30-s video clips it was given a score 2; when it happened in only one 30-s video clip, it was given score 1; and when it did not show tension of the muzzle in either 30-s video clip it received score 0. S
Tension of the mimic muscles [22,24,26]	--	There is tension of the muscles visible on the lateral aspect of the head, especially *m*. *zygomaticus* and *m*. *caninus*, but *m*. *masseter* may also be tense. When a horse showed tension of the mimic muscles in both 30-s video clips it was given a score 2; when it happened in only one 30-s video clip, it was given score 1; and when it did not show tension of the mimic muscles in either 30-s video clip it received score 0. S

Prior to the analysis of the selected 30-s video clips for each horse, five 30-s video clips were randomly selected from five different horses for training the observer to record the horses’ facial expressions. Moreover, ten other 30-s video clips were randomly selected from ten different horses to test intra-observer reliability.

### Other behaviours

Firstly, the 5-min whole body video recordings were watched twice (using normal- and fast-motion speed) by the same observer to build the ethogram of indicators to assess physical tiredness in ranch horses. Furthermore, similarly to what we did with the facial expressions, additional behaviours were selected from previous studies that assessed pain in horses [10, 31–34].

The frequencies and/or durations of the other behavioural categories were recorded using instantaneous sampling at 5-s intervals, and continuous recordings for 5-min (Table 3, [30]). The 5-min video recordings were transferred to the ELAN software (ELAN Linguistic Annotator, version 4.9.4) for coding behaviour for continuous recordings and VCL software (VCL media player, version 2.2.2) for instantaneous sampling. For these analyses, each 5-min video recording needed to be watched 8 times using normal- and slow-motion speeds in order to collect all the data.

**Table 3:** Ethogram of the 34 other behaviour variables (adapted from [10, 32–37]) and respective recording method (F = frequency and D = duration; only for “head position” and “legs supporting body” was instantaneous sampling used, for the rest of the behavioural categories continuous sampling was used).

Behavioural categories	Descriptions
Weight-shifting forelegs [10]	Supports its body weight by four legs, then shifts weight to support only three legs while the foreleg remains without load for at least 1 second. F
Resting hindleg	Supports its body weight by three legs, while resting one hindleg that remains without load (distinguishing between left and right hindleg). D
Locomotion [36]	Moves at least once with two legs, forward, backward, or sideways resulting in new position. F
Stamp high [36]	Raises the foreleg, flexes the carpus joint, and lowers quickly above carpus line onto the same spot. F
Stamp low [36]	Raises the foreleg, flexes the carpus joint, and lowers quickly below carpus line onto the same spot. F
Knocking high [36]	Raises the hindleg, flexes the tarsus joint and lowers it quickly above tarsus line onto the same spot. F
Knocking low [36]	Raises the hindleg, flexes the tarsus joint and lowers it quickly below tarsus line onto the same spot. F
Kicking at abdomen [36]	Lifts its hindleg to strike at the abdomen and hoof touches its abdomen. F
Three legs supporting body [10]	Three legs supporting the body weight and one hind leg without load. F
Paw [35]	One foreleg is lifted off the ground and extended forward followed by a dragging backward movement with the hoof digging the ground. F
Tail swishing high [32]	Moves its tail to the left or right above the line groin or moves it up at least 45 degrees and down quickly. F
Tail swishing low [32]	Moves its tail to the left or right below the line groin. F
Skin movements front [37]	Contracts the cutaneous muscle at wither’s region. F
Skin movements back [37]	Contracts the cutaneous muscle at groin’s region. F
Bodyshake [33]	Rapid, rhythmic rotation of the head, neck, and upper body along the long axis while standing with feet planted. F
Headshake [33]	Turns head either to right or left so white of eye seen. F
Head-tossing [33]	Tosses the head up or down, with or without moving the neck in the same direction simultaneously. This movement may happen in sequence, with or without alternating the direction of movement (up and down). F
Head-turning	Keeps the head towards one side (left or right) with a flexed neck, looking to a stimulus somewhere in the environment, including the observer, with one or both ears orientated forward. The horse remains a few seconds in this position before returning the head in the longitudinal axis of its body (distinguishing between left and right head turn). F and D
Touch	Touches with muzzle quickly a part of its body, may or may not rub its body with muzzle once. F
Scratch	Opens its mouth, distances its lower jaw from the upper jaw and closes its teeth, nibbling a part of its body. Or the horse touches itself with the muzzle and performs at least two movements with the muzzle rubbing it from one side to another on a part of its body. F
Rub	Part of the horse’s body touches a surface of the environment and while pressing its body to the surface drags the body part in contact with the surface from one direction to another. F
Chew	Distances the lower jaw from the upper jaw in the vertical or oblique direction, being able to perform a vertical or circulatory movement and rapidly decreases the distance between the lower jaw of the upper jaw, approaching the upper and lower teeth. This behaviour can be performed with the mouth open or closed. This movement may or may not occur together with licking. F
Lick	Exposes its tongue out of the mouth, touching or not touching its lips and returns the tongue into the mouth. This movement can occur together with chew. F
Yawn [36]	Mouth opens, head extends and rises, eyes roll and close, lower jaw rotates before mouth closes. F
Groan [34,35]	Monotone hum-like sound produced during exhalation, typically lasting up to two seconds. F
Snort [34,35]	Sound produced upon forceful quick exhalation of less than one second duration. Associated with olfactory investigation, prancing, posturing, and closed combat involving rearing, boxing, kneeling, and circling. F
Flehmen [36]	Extends the head forward, curling the upper lip. F
Head position [35]	Upper neck line is held above the wither’s line. F
	Upper neck line is in same height as the wither’s line. F
	Upper neck line is below the wither’s line. F
	Nose between the abdominal line and carpus. F
Defecation [36]	Lifts its tail, evacuating faeces. F
Urination [36]	Visible urinary stream. F

Five 5-min video recordings from previous data collection were randomly selected for training how to assess other behaviours (body view). Furthermore, ten different 5-min video recordings were randomly selected to test intra-observer reliability.

### Intra-observer reliability

Intra observer reliability was tested for facial expressions and for other behavioural variables using Weighted Kappa coefficient (*k*) considering sufficient moderate agreement (*k* ≥ 0.41) according to Landis and Koch [38]. The same observer evaluated each video twice, with at least 24 h between evaluations.

Regarding the facial expression variables, very high agreements (*k* = 0.80 to 1.0) were found for 7/18 (38.9%) variables (frequencies of blink fast and blink lower, lower lip depressor, eye partially closed, upper lip raiser, and for scores of tensions of mimic muscles and muzzle); substantial agreements (*k* = 0.60 to 0.79) for 6/18 (33.3%) of the variables (frequencies of contraction of the orbicularis muscle, and duration of lower lip relaxed, ears orientate forward, midline, backward and flattener); and moderate agreements (*k* = 0.40 to 0.59) for 4/18 (22.2%) of the variables (frequency of eye white showing, and durations of inner brow raiser and eye partially closed and nostril lift). The frequency of eye fully closed was not recorded during the reliability tests and had a low frequency during the actual data collection.

Regarding the other behaviours, very high agreements (*k* = 0.80 to 1.0) were found for the measurements of 20/31 (64.6%) of the variables (frequencies of defecation, knocking high and low, skin movements front and back, weight-shifting forelegs, scratch, touch, head-turning, lick, yawn, bodyshake, headshake, head-tossing, stamp low, paw, all head positions and three legs supporting body and durations of resting hindleg); substantial agreements (*k* = 0.60 to 0.79) for 7/31 (22.5%) of the variables (frequencies of locomotion, kicking at abdomen, tail swishing high and low, rub, chew, stamp high). Urination, flehmen, snort and groan were not recorded during the reliability tests and had a low frequency during the actual data collection.

### Physiological measurements

#### Blood sampling and analyses

Prior to starting the data collection, we collected blood samples from all horses to assess their reactions during the procedure. Nine horses from F1 performed avoidance/aggressive behaviours (e.g. attempts to bite or kick, moving the neck or body away from the handler, positioning ears backwards) during blood sampling. To reduce such reactions these horses were trained to restraint and blood sampling, the training sessions were carried out using counter-conditioning techniques [39] and cracked corn, carrots, or brown sugar as positive reinforcement (PR), according to the horse’s preference. The training process lasted on average for 20±5 continuous min/day, and the training sessions were carried out during 6±2 days per horse. The procedures adopted were described by Trindade et al. [40]. By the end of the training period, 6 ranch horses were considered “conditioned” to the blood sampling, since they did not show any avoidance behaviours and serum cortisol concentration was close to baseline (mean±SD: 4.67±0.6 mg/dL; [41]). The other three horses did not change their behaviour and were excluded from the study. The horses from F2 did not show any avoidance behaviour during the preliminary blood sampling and, thus, were not trained for blood sampling.

Blood samples were collected by venipuncture of the jugular vein intercalating between the left and right jugular every other time. For determining lactate (LA) and glucose (GL) concentrations, 4 ml of blood was collected in tubes containing Fluoride/EDTA (BD^®^, Brazil). For the level of creatine kinase (CK), aspartate aminotransferase (AST) and total protein (TP), 10 ml tubes containing clot activator and separating gel (SST II gel Advance; BD^®^, Brazil) were used, and 10 ml tubes containing heparin (BD^®^, Brazil) were used for serum cortisol (SC).

Immediately after venipuncture, all blood samples were stored in a thermal box with recyclable ice and kept in the shade until the post-workday collection was finished. After the collection period, all samples (LA, GL CK, AST, TP and SC) were centrifuged (SL-707, SoLab^®^, Brazil) at 4°C at 5,000 rpm for 5 min and, subsequently, the supernatant from the LA, CK, AST and SC samples was pipetted and transferred to labelled Eppendorf^®^ tubes and stored at a temperature of -4°C.

Total protein assays were obtained in duplicate (CV = 4.7%) by the biuret method in a semiautomatic analyzer (Labquest, Labtest^®^, Brazil); CK assays were performed by the IFCC method on a semiautomatic analyzer (Labquest, Labtest^®^, Brazil); and AST assays were determined by the UV-IFCC Kinetic method on a semiautomatic analyzer (Labquest, Labtest^®^, Brazil) using a commercial kit (Labtest^®^, Brazil). The LA and GL assays were performed by electro-enzymatic method on an automatic analyzer (YSL 1500 Sport, Yellow Springs^®^, United States) and we repeated the three first samples three times (beginning, middle and end) during the analysis (CV = 11% and 4% for lactate and glucose, respectively). Serum cortisol concentration was measured by an ELISA kit (Enzo Life Sciences Inc., Farmingdale, NY, USA) and the intra-and inter-assay coefficients of variation were 2.7% and 6.0%, respectively.

### Workload, and heart and respiratory rates

The amount of physical activity carried out by the horse during the workday was recorded by a global positioning and heart rate monitoring system (GPS-HR—Garmin Forerunner 310XT) that document the distance covered (km), exercise duration (h), and the average velocity (km/h). It also records the heart rate (beats/minute) when connected to an equine adapter kit (Equine Heart Rate Monitors System, V-Max^®^, USA). The electrodes of the digital heart rate monitor were positioned between the 3rd to the 6th thoracic intercostal space above the humeral scapular joint on the left and right sides of the withers (which were previously soaked with saline and conductive gel before electrode placement); the electrodes were attached to the animal’s body with elastic tape [27]. We recorded heart rate during the workday, immediately post-workday and 10 min post-workday to evaluate the recuperation time. During this period horses were kept with their saddle on their back in the saddling facilities. Respiratory rate was recorded before and after workday as described in Table 1, by counting respiratory movements on the equine flank or nostrils during 15 s before and immediately after the workday. The values were multiplied by four to determine the respiratory rate per minute (RR).

### Clinical examination

In the clinical examination, we assessed the body condition and lameness. We applied the body condition scores described by Wageningen UR Livestock Research [42]. Lameness was scored during the walk, while the stockperson led the horse to or from the experimental area or when going back to pasture. We used three lameness scores: (0) no evidence of irregular locomotion or lameness, (1) evidence of irregular, stiff, short locomotion; not possible to point out which leg is causing the irregular motion or lameness, and (2) evidence of lameness; clear which leg is causing the lameness [42].

### Statistical analysis

The statistical analysis was performed using RStudio (Version 1.0.143 −^©^ 2009–2016, RStudio, Inc. [43]). Descriptive statistics were carried out, and the data were presented as median and range (median; maximum-minimum) or mean and standard deviation (mean±SD). All data were tested for normality using Shapiro-Wilk test (*P*≤0.05; function “shapiro.test” of the “stats” package), and only data from CK, AST, GL and LA satisfied this criterion after being log transformed.

The complementary statistical analyses were carried out in four stages: (*i*) comparisons between pre- and post-workday; (*ii*) selection of the most powerful variables; (*iii*) evaluations of the relationship between the selected variables; and (*iv*) identifying a critical level of the most robust and feasible behavioural indicator. The pre- *vs* post-workday comparisons of CK, AST, GL and LA were made using linear mixed-effects models with repeated measures (function “lmer” of the R “lme4” package), considering sampling time as fixed effects, and each horse as a random effect. The means of sampling times were compared using the Tukey test (*P*<0.05; functions “lsmeans” and “cld” of the “lsmeans” package). The paired Wilcoxon test (function “wilcox.test” of the “stats” package) was used to analyse all other data, comparing the pre- and the immediately post-workday assessments. A probability level of *P*<0.05 was chosen as the limit for statistical significance.

In the second stage, we calculated the differences between the variables that showed significant changes between pre-and post-workday. For CK and LA (which were the only variables that had more than two post-workday assessments) we calculated the differences between the pre-workday and the12 h and the 30 min post-workday means, respectively. These were the two post-workday means with the lowest *P*-values after applying the Tukey test. Additionally, we used the Kaiser-Meyer-Olkin test (function “KMOS” of the “REdaS” package) to select the most powerful of pairs of variables that showed clear inter-dependencies (i.e., differences in frequencies of the skin movements front *vs* back, the frequency *vs* duration of eye partially closed, frequency *vs* duration of head-turning, and exercise duration *vs* distance covered). After that, we performed three Principal Components Analyses (PCA; function “princomp” of the “stats” package, and the functions “get_pca_var” and “fviz_pca_var” of the “factoextra” package), using two workload variables (exercise duration and average velocity) and the most powerful variables among those represented by the significant differences between pre- and post-workday of the facial expressions, other behaviours, and physiological variables. Based on the results of these analyses, we selected the variables that had high loading values (≥ 0.60 or ≤ -0.60) that were also associated with workload variables on dimensions with eigenvalues higher than 1, and variances higher than 20%.

Then, in the third stage, we estimated the relationships between the selected variables of workload, and those representing the differences in facial expressions, other behaviours and physiological responses by applying PCAs. Correlations between pairs of variables were then estimated using Spearman rank coefficients of correlations (*P*<0.05; function “rcorr” of the “Hmisc” package). In this way we could minimise any risk associated with multiple comparisons in our final analysis. Spearman rank coefficients of correlations were then estimated as a confirmatory analysis to assess the pair associations between the variables that were significant in the final PCA. The probability level applied in the final PCA was the same as that used in the previous PCAs.

Finally, we subjected the difference of weight-shifting between the forelegs and exercise duration to a non-hierarchical cluster analysis (functions “dist”, “hccluster” and “plot” of the “stats” package), following the "ward.D" method using “euclidean” measurement matrix distance and, then we applied the Wilcoxon test (*P* ≤ 0.05) to compare the difference of weight-shifting between the forelegs between the groups formed by the cluster analysis.

## Results

On average, horses had a work duration of 4.0±2.1 h, covering a mean distance of 18.7±4.5 km, with an average velocity of 5.4±1.5 km/h. The means of the heart rate and the maximum heart rate during workday were 91±11 and 146±25 bmp, respectively. The heart rate assessed immediately after the arrival of ranch horses in the corral and 10 min post-workday were 87±16 and 60±6 bpm, respectively. The measurements were taken with the horses tied at a post, without removing the saddle from their backs. None of the studied horses showed any sign of lameness pre- or post-workday, and all horses were scored as having moderate or good body condition.

### Comparisons between pre- and post-workday assessments

An overview of the changes in facial expressions, other behaviours, and physiological variables that decreased or increased when comparing the pre- and immediately post-workday assessments in the14 ranch horses is shown in Table 4.

**Table 4:** Overview of the facial expressions, other behaviours, and physiological variables that decreased (A) or increased (B) when comparing the pre- and immediately post-workday assessments in 14 ranch horses. Physiological parameters are presented using the 30 min and 12 h post-workday assessments for lactate and creatine kinase, respectively. Medians and ranges (maximum-minimum) or means and standard deviations (mean±SD) are presented.

**A**	**Variables that decreased post-workday**					
Groups of Categories	Variables	Pre-workday		Post-workday		
		Median	Range	Median	Range	*P*-values
Facial Expressions	Ears forward ^d^	18.5	0.0–102.1	2	0.0–35.5	0.025
	Eye white showing ^f^	3	0–20	1	0–3	0.006
	Nostril lift ^d^	4	0.0–57.3	0	0.0–37.2	0.014
Other Behaviours	Skin movement back ^f^	31	3–90	3.5	0–40	0.003
	Skin movement front ^f^	143	1–341	76.5	22–208	0.020
	Touch ^f^	5	0–18	0	0–15	0.012
	Head-turning ^d^	99	5.0–199.0	32.5	1.0–122.0	0.010
	Head-turning ^f^	10	2–35	5.5	1–19	0.035
**B**	**Variables that increased post-workday**					
Groups of Categories	Variables	Pre-workday		Post-workday		
		Median	Range	Median	Median	Range
Facial expressions	Eye partially closed ^d^	1.1	0.0–19.9	34.4	0.0–57.6	0.001
	Eye partially closed ^f^	0.5	0–3	3	0–5	0.013
	Tension of the muzzle ^s^	0	0–0	2	0–2	0.004
Other behaviours	Three legs support body ^f^	3	0–29	12.5	1–60	0.003
	Weight-shifting foreleg ^f^	0.5	0–8	5	0–18	0.017
		Mean	SD	Mean	SD	*P*-values
Physiology	Respiratory rate (mpm)	9.7	25.5	21.4	4.6	0.000
	Cortisol (mg/dl)	7.0	23.5	14.1	5.6	0.001
	Lactate (mmol/L)	0.706	0.1	0.978	0.2	0.000
	Creatine Kinase (mmol/L)	271.1	60.4	339.7	84.3	0.040

^d^ = duration (s),

^f^ = frequency and

^s^ = score.

Three variables of facial expressions (durations of ears forward and nostril lift, and frequency of eye white showing) decreased post-workday, as did five variables of other behaviours (frequencies of skin movements back and front, touch, head turning, and duration of head-turning), as shown in Table 4A. Duration and frequency of eye partially closed, score of tension in the muzzle, and frequencies of three legs supporting the body and weight-shifting between forelegs increased after the workday (Table 4B).

Similarly, most of the physiological responses (respiratory rate, cortisol and creatine kinase at 12 h post-workday) also increased after the workday (Table 4B), as did the lactate concentrations, which were higher for all measurements taken post-workday, at 15 (0.922±0.2 mmol/L; *P* = 0.004), 30 (0.978±0.2 mmol/L; *P* = 0.000), and 60 min (0.977±0.2 mmol/L; *P* = 0.000), compared to pre-workday (0.706±0.1 mmol/L).

### Relationships between body language, physiological and workload variables

The first dimension of the PCA performed using the workload variables and the facial expressions, showed high loadings for duration of eye partially closed, exercise duration, ears forward and average velocity. At the second dimension of this PCA only one variable (eye white showing) had a high loading (Table 5A). The second PCA, performed using workload for the horse and the other behaviours, showed high loadings in dimension 1 for weight-shifting between forelegs, skin movements front, touch, exercise duration, and average velocity, but in the second dimension only head-turning duration had a high loading (Table 5B). Finally, the first dimension of the third PCA, carried out using workload variables for the horse and the physiological variables, showed high loadings for exercise duration and average velocity and cortisol, while the second dimension had high loading values for creatinase (Table 5C).

**Table 5:** Variables’ loadings, eigenvalues and variances of the workload variables and the “differences” of the (A) facial expressions, (B) other behaviours and (C) physiological variables performed by Principal Components Analysis assessed in 14 ranch horses.

**A) Facial expressions**			
**Variables**	**Dimension 1**	**Dimension 2**	
Eye partially closed ^d^	**0.63**	-0.39	
Eye white showing ^f^	-0.00	0.85	
Nostril lift ^d^	-0.07	0.57	
Ears forward ^d^	**-0.74**	-0.16	
Tension of the muzzle ^s^	0.55	0.57	
Exercise duration	**0.89**	0.02	
Average velocity	**-0.93**	0.19	
Eigenvalues	2.9	1.6	
Variance (%)	42.01	23.06	
**B) Other behaviours**			
**Variables**	**Dimension 1**	**Dimension 2**	
Weight-shifting forelegs ^f^	**-0.72**	0.40	
Skin movements front ^f^	**-0.89**	-0.17	
Touch ^f^	**-0.79**	-0.32	
Head-turning ^d^	-0.23	-0.86	
Three legs supporting body ^f^	0.57	0.40	
Exercise duration	**-0.86**	0.40	
Average velocity	**0.91**	-0.23	
Eigenvalues	3.9	1.4	
Variance (%)	56.10	20.59	
**C) Physiological variables**			
**Variables**	**Dimension 1**	**Dimension 2**	**Dimension 3**
Cortisol	**-0.62**	0.26	0.37
Respiratory rate	-0.08	-0.45	0.84
Lactate	-0.31	-0.57	-0.58
Creatine kinase	-0.17	-0.90	0.03
Exercise duration	**0.89**	-0.20	-0.06
Average velocity	**-0.95**	0.03	-0.07
Eigenvalues	2.2	1.4	1.2
Variance (%)	37.62	24.44	20.17

^d^ = duration (s),

^f^ = frequency and

^s^ = score. Loadings in bold indicate high associations with the dimension.

**Fig 2:**
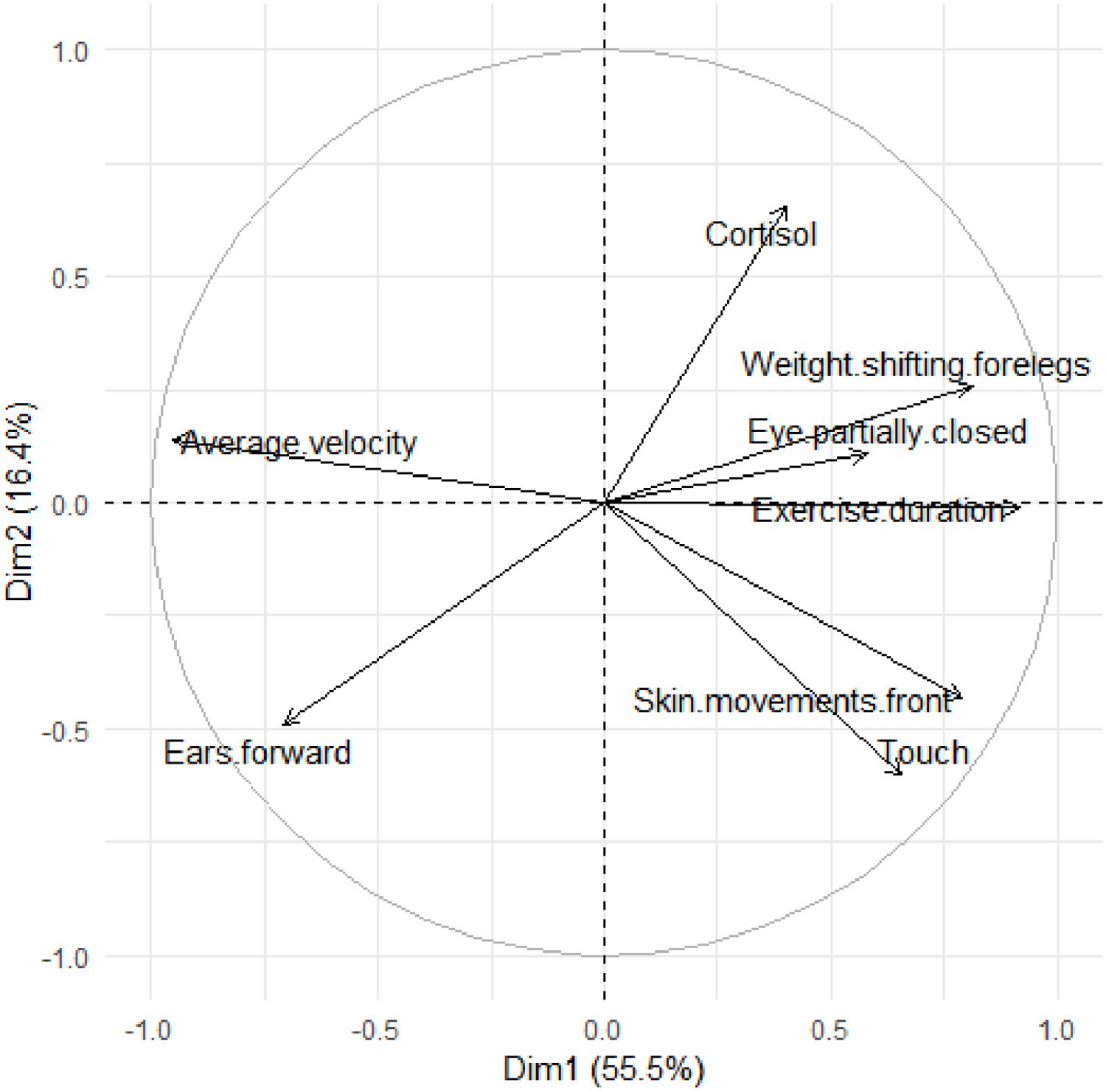
Projections of the loading values in the two dimensions of the Principal Component Analysis performed with the selected variables of the workload variables (exercise duration and average velocity), and the pre- and post-workday differences of facial expressions (eye partially closed, eye white showing and ears forward), other behaviours (weight-shifting forelegs, skin movements front, and touch) and physiological variable (cortisol) assessed in 14 ranch horses.

In the final (fourth) PCA, performed with the selected variable set (Fig 2), the first dimension explained 55.47% of the total variance (eigenvalue = 4.4) and showed positive associations with exercise duration (0.91), differences in weight-shifting between the forelegs (0.81), skin movements front (0.78), and touch (0.66); and negative associations with average velocity (-0.96) and difference in ears forward (-0.71). The second dimension explained 16.44% of the total variance (eigenvalue = 1.3) and showed negative associations with cortisol (0.66) and difference in touch (-0.60). As further confirmation of the associations between the eight selected variables that were significant in this fourth PCA, we estimated the Spearman rank coefficients of correlation, as shown in Table 6.

**Table 6:** Significant Spearman rank coefficients of correlation between the workload variables (exercise duration and average velocity), and the pre- and post-workday differences in facial expression (ears forward), other behaviours (weight-shifting forelegs and touch), and physiological variable (cortisol) of 14 ranch horses.

Variables		*Rho*-value	*P*-value
Exercise duration	Cortisol	0.62	0.0180
Exercise duration	Weight-shifting forelegs	0.64	0.0138
Average velocity	Skin movements front	-0.58	0.0289
Average velocity	Touch	-0.73	0.0030
Average velocity	Eye partially closed	-0.56	0.0380
Ears forward	Weight-shifting forelegs	-0.75	0.0019
Skin movements front	Touch	0.77	0.0012

**Fig 3:**
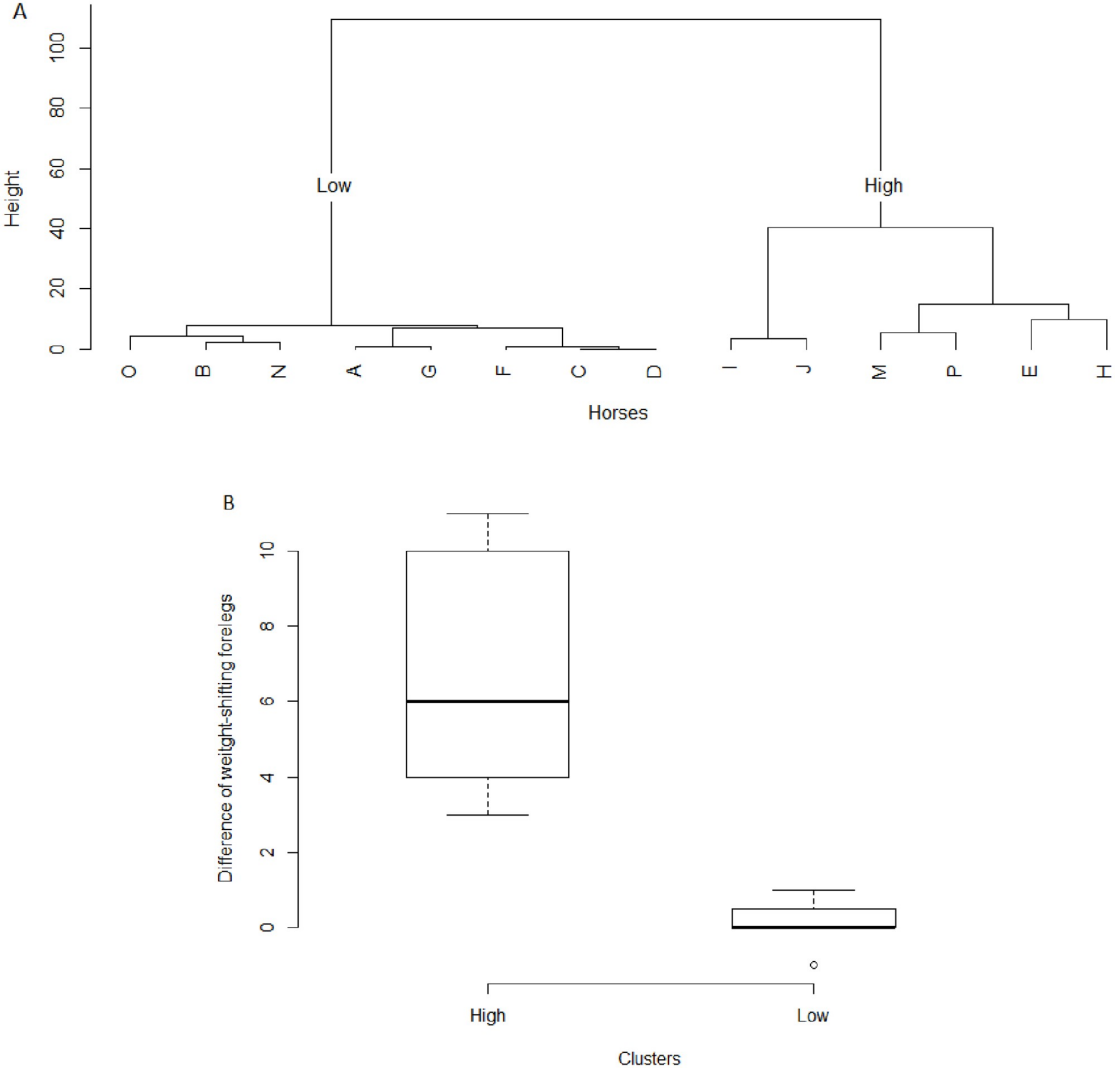
Dendrogram generated through non-hierarchical cluster analysis applied to the difference of weight-shifting forelegs and exercise duration; showing the formation of two clusters, LOW and HIGH (A). Data were assessed pre- and post-workday in 14 ranch horses. Medians of difference of weight-shifting between the forelegs (B) for each cluster (LOW and HIGH) are presented in boxplots.

The non-hierarchical cluster analysis separated the horses into two groups (LOW and HIGH), and the difference of weight-shifting between the forelegs was statistically different between the groups (Fig 3). The median (range) of weight-shifting forelegs for LOW pre- and post-workday were respectively 0.5 (0–8) and 1 (0–8), and for HIGH 0.5 (0–7) and 7 (5–18). The horses’ age, duration of work and frequency of weight-shifting forelegs are shown in Table 7.

**Table 7:** Age of the 14 ranch horses and respective medians of the frequencies of weight-shifting forelegs assessed before and after a workday.

Period of work	Horse	Age	Frequencies of Weight-Shifting Forelegs	
			Pre-Workday	Post-Workday
Half day	A	7	0	1
	C	8	5	5
	D	8	0	0
	G	9	0	1
	E	15	1	5
	F	16	0	0
	H	16	0	7
	N	18	8	8
	B	20	1	1
	O	27	2	1
Entire day	P	6	0	5
	I	11	7	18
	M	14	4	7
	J	24	0	10

## Discussion

To the best of our knowledge, this is the first study examining the use of body language as an indicator of the degree of ranch horses’ physical tiredness and we found clear differences in the body language of ranch horses after a routine of working day. We identified some specific body language changes indicative of an increase in resting, a decrease in attention to the surroundings and fewer movements to avoid flies after a workday that, from our perspective, have potential to be used to assess physical tiredness in horses. Of particular interest, because of the ease with which it can be assessed on the farm and generalized to other situations, was the increased frequency of shifting weight between the forelegs which, we suggest, may be a useful indicator of physical tiredness in horses.

### Comparisons of the body language and physiological variables between pre- and post-workday assessments

We recorded behavioural categories of body language (including facial expressions and other behaviours) that we deemed relevant to assess physical tiredness in horses. Some of these have been used previously for assessing pain in horses. We expected that the assessed variables (n = 52) would change after a workday, but this was only partially confirmed since less than one-third of them actually changed. One plausible explanation for this may be that the ranch horses performed a submaximal aerobic exercise of low intensity and long duration, which did not cause acute physical exhaustion, rhabdomyolysis or overtraining. This is supported by the workload variables and the values of lactate plasmatic concentration, creatine kinase activity, respiratory and heart rates observed in our study compared to the values presented by Hodgson et al. [44] and Teixeira-Neto et al. [45]. Another reason might be that the measurements were taken at the end of the workday and not directly in association with any specific effort (e.g. galloping for a prolonged period). If this activity was early in the day, some of the variables may have returned to baseline levels by the time the horse ended the workday.

Five body language variables increased after a workday (duration and frequency of eye partially closed, tension of the muzzle, frequencies of three legs supporting body and weight-shifting foreleg), and most of them (except tension of the muzzle) were described as prevailing during resting by McDonnell [35] and Williams *et al*. [46]. From the other eight variables that decreased after the workday, we associated four of them (duration of ears forward, frequency of eye white showing, and frequency and duration of head-turning) with attention to the surroundings, and three of them (touch, skin movements front and back) have previously been associated with movements to avoid flies [35].

Fleming et al. [47] applied qualitative behaviour assessment (QBA) at the regular inspections during endurance competitions, and scored horses as more “alert”, “curious” and “excited” pre-endurance ride, while at the end they were scored as more “tired”, “lazy” and “sleepy”; and their results [47] support our findings that ranch horses showed more resting behaviours, and less attention and movements after physical exercise, indicating some degree of physical tiredness.

However, the use of these body language categories as indicators of physical tiredness is not clear cut, since some of them can also be displayed by horses when experiencing very different states. For example, eye partially closed can be observed when horses rest [48], or when they experience pain after castration and acute colic surgeries [22, 25], or even during competition [26]. However, eye partially closed was not observed when pain was induced in horses [24]. The authors argued that it was not possible to discriminate between whether horses were in pain or whether eyes partially closed was a consequence of being physically tired after the surgery. Increases in the frequencies of weight-shifting forelegs, three legs supporting body and middle head position were previously reported for horses in pain [101, 49–51], and also during resting [35, 46].

The changes observed in the duration of nostril lift, and frequencies of upper lip raiser, lower lip depressor, touch and skin movements (front and back) have not previously been reported as being indicators of either pain or resting in horses. The nostril lift, upper lip raiser and lower lip depressor were described by Wathan et al. [29], but we did not find any information about their meaning. We have two possible explanations for the increase in lip movements, these are: (*i*) discomfort in the mouth area probably due to a dry mouth; (*ii*) or discomfort arising from pulling on the reins by the stock person.

In our study, the changes in the variables assessed pre- and post-workday did not help us to distinguish between physiological responses to aerobic low-intensity exercise, pain or other internal states, related to the use of the horse. Firstly, because an increase in the physiological indicators is expected after physical exercise [52, 53], and when horses feel pain [8]. Moreover, the decrease in heart rate (assessed immediately after workday and 10 min later) presumably indicated that the horses were showing signs of recovery from exercise, although it would also have been useful to have recorded time for heart rate and respiratory rate to return to resting values after the workday. The creatine kinase activity increased after a workday, but the values were very close to the maximum activity value within the normal range [54]. This increase in the CK activity and the facial tension post-workday could nevertheless be interpreted as slight pain induced by transitory changes in sarcolemma permeability [55], that did not produced lameness.

In summary, based on the results presented above, we suggest that it is most realistic to assume that the changes observed in the body language and physiological variables reflect that some horses were tired or very tired, but without any of the deleterious consequences of physical activity. Nevertheless, the ranch horses were apparently experiencing a combined state of post-aerobic effort and slight soreness after a working day. We identified an apparent increase in the body language of resting, a decrease in attention to the surroundings and movements to avoid flies after a workday that could be attributed to the horses’earlier aerobic effort.

### Relationships between body language, physiological and workload variables

We observed relationships among some indicators of workload (exercise duration and average velocity), body language (eye partially closed, ears forward, weight-shifting forelegs, skin movements front, and touch) and stress (cortisol). The body language and stress indicators were selected during the second stage of the statistical analysis that identified the most powerful variables associated with the workload indicators. It is important to note that the workload variables used herein were adapted from a review paper [56] addressing the subject, but that they are summed or averaged over the whole work period.

We did not find any previous study assessing the relationships between weight-shifting forelegs, touch, ears forward or skin movements and physiological or workload variables in exercising horses. The only study that did assess them was carried out by Rietmann et al. [32], who reported an increase in the frequency of weight-shifting forelegs and an increased concentration of serum cortisol in horses with acute or chronic laminitis, but they did not investigate correlations. In our study, the ranch horses did not show any evidence of lameness before or after a workday, thus we can discard the possibility that they faced chronic or acute pain in their hooves or legs during the study, even if we earlier suggest they may experience some slight soreness post workday. Therewith, the positive correlation between weight-shifting forelegs and exercise duration does seem to suggest that ranch horses faced the consequences of low intensity aerobic exercise after the workday. This supports our previous findings that horses in our study were expressing the body language of resting post-workday.

The three body language variables (duration of ears forward, frequencies of skin movements front and touch) that were significantly correlated with workload variables are difficult to record under field conditions. This is because horses change ear position very quickly and the skin movements are also produced by muscle contractions, which are very frequent and fast. In addition, it is expected that horses touch their bodies when trying to repel flies, so the frequency of touch probably depends on the number of flies and would be an unreliable indicator of physical tiredness in different environmental conditions.

In summary, based on the results obtained through the second, third and fourth stages of our statistical analysis, we concluded that an increase in the frequency of weight-shifting between the forelegs has potential to be used as a practical body language indicator of physical tiredness in horses. Since it was strongly associated with the workload variables, separated the horses in the non-hierarchical cluster analysis, is easy to record under field conditions and not dependent on the environmental conditions. From a practical perspective, we propose that a frequency of weight-shifting forelegs higher than 7 per 5 min (median of HIGH cluster) has potential to signal some degree of physical tiredness in horses.

It is usually assumed that the workday routine of ranch horses on beef cattle farms in Brazil is a source of physical stress, mainly because the workdays are too long and intense, occurs under high air temperature and humidity, and often without any time for resting. However, this is not always the case, since in some farms, as is the case of F2 in this study, the horses work only for a half period of the day. Thus, the impact of reducing the daily period of work on horse effort is something that should be addressed in further and more controlled studies. These studies should involve standardizing, for example, the type and length of exercise, and assessing two or more workloads with different physical effort intensities as well as evaluating over time after the exercise. Additionally, the horses’ age must also be controlled, since it is possible that old horses are more susceptible to increase the frequency of weight-shifting forelegs than the young ones. It is worth mentioning that most of the facial expressions and other behaviours analysed in this study had previously been used in association with pain assessment. The validity of the frequency of weight-shifting forelegs as an indicator of physical tiredness in horses therefore still needs to be confirmed by future studies in horses known to be free of any pain.

Our findings can help people identify body language signals for physical tiredness in ranch horses before they reach exhaustive disease syndrome, acute exertional rhabdomyolysis or overtraining post-exercise. Training stockpeople to recognise these signals could be part of a set of good handling practices that considers a proper workload and a training program for horses, preventing deleterious effects of excessive physical exercise and promoting the welfare of ranch horses.

## Conclusions

The results partially support our hypotheses that facial expresssions and other behaviour variables change after a workday, and that some of these variables are associated with workload variables and physiological responses. This was despite the fact that the measurements were taken at the end of the workday and not in direct association with the specific time of the activity during the workday. Ranch horses showed an increased body language of resting and a decrease in both attention to their surroundings and movements to avoid flies after a workday. Since it is less influenced by the environment, the frequency of weight-shifting forelegs has potential to be a useful indicator of physical tiredness (possibly with some associated pain) in horses, but this must be confirmed by further studies, which should be carried out under more controlled conditions. Our study represents only a first step towards the future development of a composite behaviour scale to evaluate physical tiredness in horses. Additionally, the results presented in our study can help people identify behavioural traits of physical tiredness in horses before reaching acute physical exhaustion, rhabdomyolyse or overtraining. The identification of behavioural indicators of physical tiredness can improve ranch horses’ welfare, as well as the welfare of horses used in other activities.

## Supporting information

S1 Appendix (DOCX)

S1 Dataset (XLSX)
