# Supplementary material for: Effect of work on body language of ranch horses in Brazil
Source: PLoS One. 2020 Jan 28;15(1):e0228130. doi: 10.1371/journal.pone.0228130 (PMC6986720; doi:10.1371/journal.pone.0228130)
Supplement: S1 Appendix — (DOCX) [file pone.0228130.s001.docx]

S1 Appendix: **Hematologic profile (mean±SD) of 14 ranch horses**. RBC = red blood count; MCV = mean corpuscular volume; MCHC = mean corpuscular hemoglobin concentration; MCH = mean corpuscular hemoglobin; WBC = white blood cell; reference values according to Zobba et al. [41].

| **Horses** | **RBC**  **(6.80 – 12.90 10^6^/µL)** | **Hemoglobin**  **(11 - 19 g/dL)** | **Hematocrit**  **(32 – 53%)** | **MCH**  **(12.3 – 19.9 pg)** | **MCHC**  **(31.0 – 38.6 g/dL)** |
| --- | --- | --- | --- | --- | --- |
| O | 7.34 | 11 | 34 | 15 | 32.4 |
| N | 6.91 | 11.8 | 35.3 | 17.1 | 33.4 |
| G | 6.45 | 10.6 | 32.7 | 16.4 | 32.4 |
| H | 6.27 | 10.1 | 30.9 | 16.1 | 32.7 |
| A | 7.51 | 12.2 | 37 | 16.2 | 33 |
| B | 7.33 | 11.7 | 36.6 | 16 | 32 |
| D | 6.73 | 10.3 | 31.8 | 15.3 | 32.4 |
| C | 7.32 | 10.8 | 33.9 | 14.8 | 31.9 |
| E | 9.83 | 15.2 | 48.5 | 15.5 | 31.3 |
| F | 6.73 | 11.6 | 36.1 | 17.2 | 32.1 |
| I | 7.18 | 10.8 | 33.7 | 15 | 32 |
| J | 6.9 | 12.5 | 37.5 | 18.1 | 33.3 |
| M | 7.91 | 12.3 | 37 | 15.5 | 33.2 |
| P | 7.87 | 12.1 | 36.7 | 15.4 | 33 |
| Mean±SD | 7.31±0.57 | 11.64±0.90 | 35.84±2.65 | 15.97±0.76 | 32.51±0.51 |

| **Horses** | **MCV**  **(37 – 58 fL)** | **WBC**  **(5.4 – 14.3 10^3^/µL)** | **Rods neutrophils (0.00 – 0.40 10^9^/L)** | **Segmented neutrophils (2.26 – 8.50 10^9^/L)** | **Lymphocytes**  **(1.50 – 7.70 10^9^/L)** |
| --- | --- | --- | --- | --- | --- |
| O | 46.3 | 8.9 | 0.00 | 5.62 | 3.37 |
| N | 51.1 | 7.8 | 0.00 | 7.31 | 3.85 |
| G | 50.7 | 8.6 | 0.00 | 6.51 | 3.95 |
| H | 49.3 | 8.5 | 0.00 | 7.65 | 3.29 |
| A | 49.3 | 8.3 | 0.00 | 4.46 | 6.87 |
| B | 49.9 | 12.1 | 0.00 | 4.30 | 3.39 |
| D | 47.3 | 11.8 | 0.00 | 4.32 | 3.73 |
| C | 46.3 | 10.8 | 0.00 | 4.72 | 4.17 |
| E | 49.3 | 11.3 | 0.00 | 4.51 | 3.45 |
| F | 53.6 | 9.7 | 0.00 | 6.70 | 3.20 |
| I | 46.9 | 11.9 | 0.00 | 4.71 | 3.28 |
| J | 54.3 | 10.9 | 0.00 | 5.50 | 3.03 |
| M | 46.8 | 12 | 0.00 | 4.75 | 3.08 |
| P | 46.6 | 12.3 | 0.00 | 4.07 | 3.74 |
| Mean±SD | 49.12±2.08 | 10.35±1.47 | 0.00±0.00 | 5.37±1.01 | 3.74±0.55 |

| **Horses** | **Eosinophils**  **(0.10 – 1.00 10^9^/L)** | **Basophils**  **(0.00 – 1.00 10^9^/L)** | **Monocytes**  **(0.10 – 1.00 10^9^/L)** |
| --- | --- | --- | --- |
| O | 2.02 | 0.00 | 0.22 |
| N | 1.28 | 0.00 | 0.38 |
| G | 0.93 | 0.00 | 0.23 |
| H | 0.71 | 0.00 | 0.12 |
| A | 0.36 | 0.00 | 0.36 |
| B | 0.33 | 0.00 | 0.25 |
| D | 0.34 | 0.00 | 0.08 |
| C | 0.09 | 0.00 | 0.28 |
| E | 0.71 | 0.00 | 0.18 |
| F | 0.31 | 0.00 | 0.10 |
| I | 0.25 | 0.00 | 0.17 |
| J | 0.46 | 0.00 | 0.18 |
| M | 0.25 | 0.00 | 0.25 |
| P | 0.16 | 0.00 | 0.16 |
| Mean±SD | 0.59±0.39 | 0.00±0.00 | 0.21±0.07 |
